# Supplementary figures and images for: Production, Characterization, and In Vitro and In Vivo Studies of Nanoemulsions Containing St. John’s Wort Plant Constituents and Their Potential for the Treatment of Depression
Source: Pharmaceuticals (Basel). 2023 Mar 26;16(4):490. doi: 10.3390/ph16040490 (PMC10141068; doi:10.3390/ph16040490)

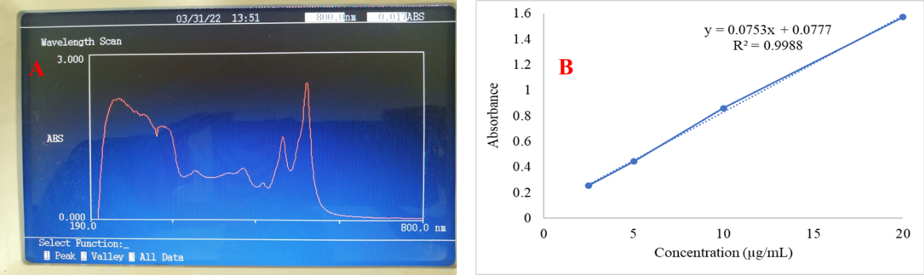

Supplement: Supplementary file 1 [file pharmaceuticals-16-00490-s001.zip › Figure S1. (A) Determination of Lambda max for hypericin by scanning the drug sample in the range of 190 to 800nm and (B) the linearity behavior of the drug at 243nm.png]

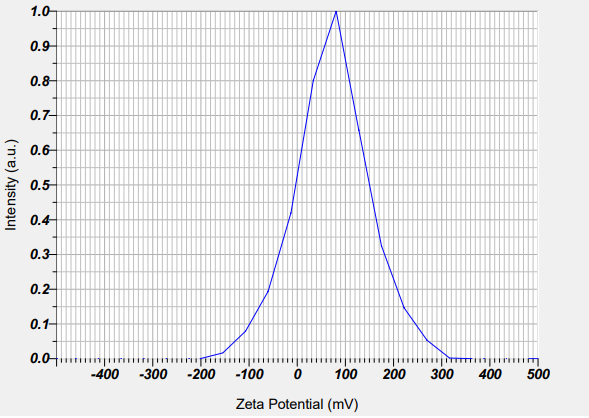

Supplement: Supplementary file 1 [file pharmaceuticals-16-00490-s001.zip › Figure S2A. Illustrating zeta potential on the particle surface(F1 formulation), making it well dispersed formulation..png]

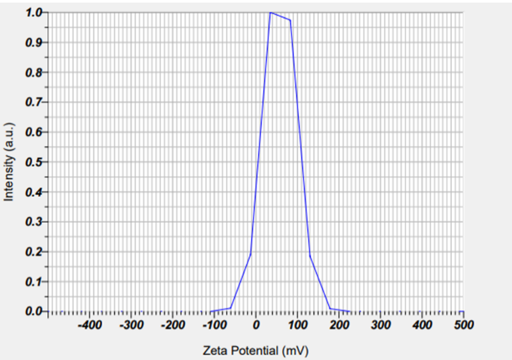

Supplement: Supplementary file 1 [file pharmaceuticals-16-00490-s001.zip › Figure S2B. Illustrating zeta potential on the particle surface(F2 formulation), making it well dispersed formulation..png]

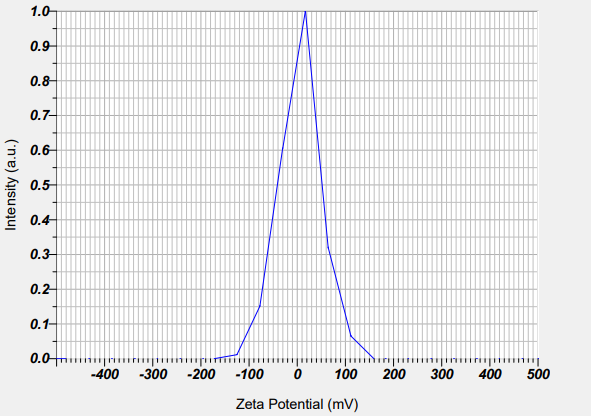

Supplement: Supplementary file 1 [file pharmaceuticals-16-00490-s001.zip › Figure S2C. Illustrating zeta potential on the particle surface(F3 formulation), making it well dispersed formulation..png]

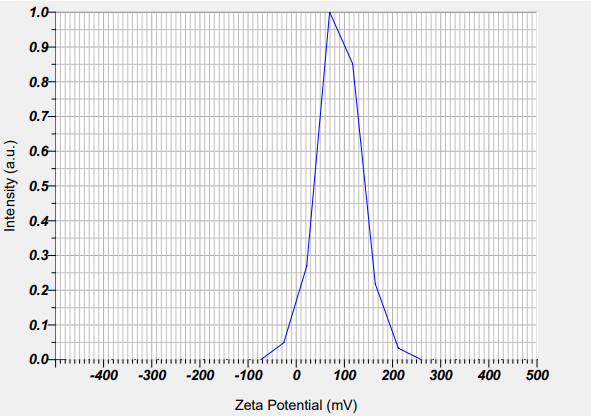

Supplement: Supplementary file 1 [file pharmaceuticals-16-00490-s001.zip › Figure S2D. Illustrating zeta potential on the particle surface(F4 formulation), making it well dispersed formulation..png]

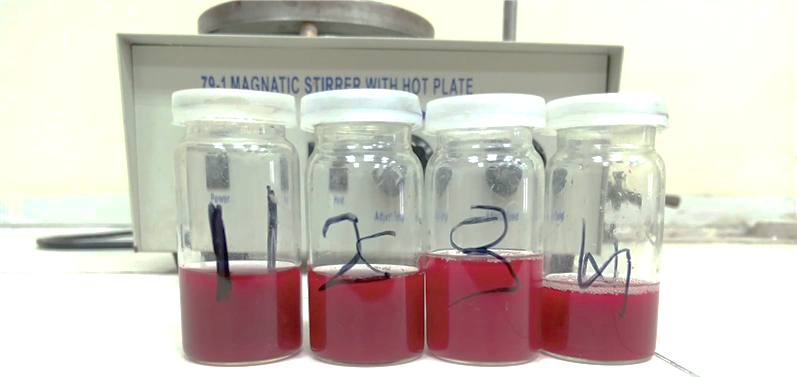

Supplement: Supplementary file 1 [file pharmaceuticals-16-00490-s001.zip › Figure S4. Prepared hypericin nanoemulsion.png]
